# Ability of Ultrasonography in Detection of Different Extremity Bone Fractures; a Case Series Study

**Published:** 2017-01-09

**Authors:** Farzad Bozorgi, Massoud Shayesteh Azar, Seyed Hossein Montazer, Aroona Chabra, Seyed Farshad Heidari, Alireza Khalilian

**Affiliations:** 1 Department of Emergency Medicine, Faculty of Medicine, Mazandaran University of Medical Sciences, Sari, Iran.; 2 Orthopedic Research Center, Faculty of Medicine, Mazandaran University of Medical Sciences, Sari, Iran.; 3 Student Research Committee, Faculty of Pharmacy, Mazandaran University of Medical Sciences, Sari, Iran.; 4 Department of Statistics, Faculty of Medicine, Mazandaran University of Medical Sciences, Sari, Iran.

**Keywords:** Ultrasonography, radiography, fractures, bone, diagnosis, emergency service, hospital

## Abstract

**Introduction:**

Despite radiography being the gold standard in evaluation of orthopedic injuries, using bedside ultrasonography has several potential supremacies such as avoiding exposure to ionizing radiation, availability in pre-hospital settings, being extensively accessible, and ability to be used on the bedside. The aim of the present study is to evaluate the diagnostic accuracy of ultrasonography in detection of extremity bone fractures.

**Methods:**

This study is a case series study, which was prospectively conducted on multiple blunt trauma patients, who were 18 years old or older, had stable hemodynamic, Glasgow coma scale 15, and signs or symptoms of a possible extremity bone fracture. After initial assessment, ultrasonography of suspected bones was performed by a trained emergency medicine resident and prevalence of true positive and false negative findings were calculated compared to plain radiology.

**Results:**

108 patients with the mean age of 44.6 ± 20.4 years were studied (67.6% male). Analysis was done on 158 sites of fracture, which were confirmed with plain radiography. 91 (57.6%) cases were suspected to have upper extremity fracture(s) and 67 (42.4%) to have lower ones. The most frequent site of injuries were forearm (36.7%) in upper limbs and leg (27.8%) in lower limbs. Prevalence of true positive and false negative cases for fractures detected by ultrasonography were 59 (64.8%) and 32 (35.52%) for upper and 49 (73.1%) and 18 (26.9%) for lower extremities, respectively. In addition, prevalence of true positive and false negative detected cases for intra-articular fractures were 24 (48%) and 26 (52%), respectively.

**Conclusion:**

The present study shows the moderate sensitivity (68.3%) of ultrasonography in detection of different extremity bone fractures. Ultrasonography showed the best sensitivity in detection of femur (100%) and humerus (76.2%) fractures, respectively. It had low sensitivity in detection of in intra-articular fractures.

## Introduction

Orthopedic injuries, including bone fractures are widespread and cause a large portion of emergency department (ED) visits. Among them, extremity injuries are frequent and result in approximately 3.5% of annual ED visits all over the world (1). Plain radiography is the ordinary standard of care for evaluation and diagnosis of long bone fractures (2). However, it could result in unnecessary radiation exposure to patients, especially children with tissues sensitive to radiation and pregnant women (3, 4). The risk of transferring critical patients to the radiography unit is another concern and it may not even be available in some areas (2, 5). Therefore, finding safer, economic, and readily available alternative imaging techniques is a matter of interest. The use of ultrasonography in both ED and pre-hospital settings has increased significantly over the last decade (6, 7). Despite radiography being the gold standard in evaluation of orthopedic injuries, using bedside ultrasonography has several potential supremacies such as avoiding exposure to ionizing radiation, availability in pre-hospital settings, being extensively accessible and affordable, and ability to be used on the bedside (2, 5, 8-10). It has been shown to be able to recognize occult fractures indiscernible on plain radiographs (11, 12). Ultrasonography can be performed during clinical examination, which may help in more rapid and accurate decision making (13, 14). The aim of the present study is to evaluate the diagnostic accuracy of ultrasonography in detection of extremity bone fractures.

## Methods

***Study design***

This study is a case series study, which evaluates the diagnostic accuracy of ultrasonography in detection of extremity bones fractures. The duration of the study was one year, from June 2014 to May 2015, and trauma patients presenting to the ED of Imam Khomeini Hospital, Sari, Iran, complaining of painful extremities were evaluated. The protocol of study was approved by the local ethics committee of Mazandaran University of Medical Sciences and authors adhered to principles of Helsinki declaration and confidentiality of patient information during the study period. Written informed consent was obtained from all patients before inclusion in the study.

***Participants***

Multiple blunt trauma patients, who were 18 years old or older, had stable hemodynamic, Glasgow coma scale 15, and signs or symptoms of a possible extremity bone fracture were prospectively enrolled. Patients, who had been carried to the operating room emergently, had decreased level of consciousness and evidence of open fractures were excluded. 

***Procedure***

An emergency medicine physician, who completed a 3-months musculoskeletal ultrasonography educational course, was responsible for initial examination and performing bedside ultrasonography for all of the participants. He performed bedside ultrasonography on upper and lower extremity bones with particular punctuality over the areas with maximum pain or tenderness. Ultrasonography was performed in longitudinal and transverse planes in supine position prior to obtaining plain radiographs. A portable ultrasonography device (sonoACE R3 System, Samsung, South Korea) with a high frequency (10 to 15 MHz) broadband linear array transducer was used for screening bone fractures. Considering the large muscle bulk, ultrasonography of femur bones was performed with deep array transducer. Abnormalities of the cortex including a break, step-off, or discontinuity of the cortical margin at the fracture site were considered as ultrasonographic signs of fracture. After initial assessment of patients according to Advanced Trauma Life Support (ATLS) guidelines, ultrasonography of long bones suspected to fracture was done and in cases of positive findings, reduction, traction, and splinting was done and the patients were referred to the radiography unit for imaging (Anterior-posterior (AP), lateral and oblique views (if indicated)). After performing standard radiography, radiography images were interpreted by an orthopedic specialist blinded to ultrasonography and clinical findings. In cases with high clinical suspicion for fracture, whose findings of plain radiography was uncertain, computed tomography (CT) scan was performed. Demographic data as well as ultrasonography and radiography findings were gathered using a predesigned checklist.

***Statistical analysis***

Statistical analyses were carried out with SPSS version 18. Qualitative data were presented as frequency and percentage, and quantitative ones as mean ± standard deviation. Prevalence of true positive and false negative results of ultrasonography in detection of extremity fractures compared to radiography (as the gold standard) was calculated.

**Table 1:** Characteristics of fractures in studied patients

**Variables**	**Frequency (%)**
**Upper extremity**	
Humerus	21 (13.30)
Radius	40 (25.31)
Ulna	18 (11.40)
Metacarpus	12 (7.6)
**Lower extremity**	
Femur	30 (19.00)
Tibia	23 (14.55)
Fibula	11 (6.97)
Metatarsus	3 (1.8)
**Location**	
Proximal	37 (23.4)
Middle	42 (26.5)
Distal	79 (50.0)
**Side**	
Left	70 (44.3)
Right	88 (55.7)
**Number **	
1	73 (67.6)
≥ 2	35 (32.4)
**Intra-articular**	
Yes	50 (31.6)
No	108 (68.4)

**Table 2:** Characteristics of ultrasonography in detection of different extremity bone fractures

**Fracture site**	**True positive n (%)**	**False Negative n (%)**
**Humerus**	16 (76.2)	5 (23.8)
**Radius**	29 (72.5)	11 (27.5)
**Ulnar**	9 (50)	9 (50)
**Metacarpus**	5 (41.7)	7 (58.3)
**Femur**	30 (100)	0 (0)
**Tibia**	10 (43.5)	13 (56.5)
**Fibula**	8 (72.7)	3 (27.3)
**Metatarsus**	1 (33.3)	2 (66.4)

## Results

108 patients with the mean age of 44.6 ± 20.4 years were studied (67.6% male). Neither radiography nor ultrasonography showed any fractures in three patients, but considering continuous pain, tenderness and limited range of motion, they underwent CT scan, which confirmed fracture of tibial plateau, distal femur, and radius head. 

Finally, analyses were done on 158 sites of fracture, which were confirmed by plain radiography. Table-1 shows the type and distributions of fractures. 91 (57.6%) cases were suspected to have upper extremity fracture(s) and 67 (42.4%) to have lower ones. The most frequent sites of injury were forearm (36.7%) in upper limbs and leg (27.8%) in lower limbs. 99 (62.7%) cases of fracture were dislocated and 50 (31%) were intra-articular. Table 2 shows characteristics of ultrasonography in detection of different upper and lower extremity bone fractures. Prevalence of true positive and false negative detected cases for fractures by ultrasonography were 59 (64.8%) and 32 (35.52%) for upper and 49 (73.1%) and 18 (26.9%) for lower extremities, respectively. In addition, prevalence of true positive and false negative detected cases for intra-articular fractures were 24 (48%) and 26 (52%), respectively.

## Discussion

The present study showed the moderate sensitivity (68.3%) of ultrasonography in detection of different extremity bone fractures. Ultrasonography showed the best sensitivity in detection of femur (100%) and humerus (76.2%) fractures, respectively. It had low sensitivity in detection of in intra-articular extremity bone fractures. 

Ultrasonography application is rising in all aspects of diagnostic studies. It seems to be secure and reliable, and is being used increasingly for diagnostic evaluations in ED (15). Previous studies have shown that bedside ultrasonography by trained clinicians is as impressive as radiography for diagnosis of long bone fractures. Patel et al. showed that ultrasonography is as useful as radiography for diagnosis of long bone fractures, and has a high sensitivity for diagnosis of such fractures(16). Haddad-Zebouni et al. showed the high accuracy of bedside ultrasonography for diagnosis of bone fractures and recommended ultrasonography for this propose(17). Hübner et al. also found that diagnosis of long bone fractures using ultrasonography is accurate in majority of cases (8). Some studies have shown that ultrasonography is more useful than radiography in diagnosis of fracture in some cases that perhaps were not detected in radiographic imaging such as hip, clavicle, knees and feet (12, 16). In contrast, Bolandparvaz et al. showed that ultrasonography was not reliable and adequate for diagnosis of long bone fractures(18). 

Majority of studies in this regard have been conducted on pediatrics population (8, 17, 19). Bolandparvaz et al. and Marshburn et al. concluded that ultrasonography is reliable only when an injury cannot be detected by radiography and showed that specificity of ultrasonography is not high enough for confirmation of long bone fractures in adults (5, 18). Based on the results of the present study, it seems that, ultrasonography could not be considered as an accurate screening tool for detection of extremity fractures in the adult population. More studies are required to infer the role of ultrasonography in detection of bone fractures in trauma guidelines. Low sample size and lack of control group are two important limitations of the present study, which undermine the generalizability of the results.

## Conclusion

The present study shows the moderate sensitivity (68.3%) of ultrasonography in detection of different extremity bone fractures. Ultrasonography showed the best sensitivity in detection of femur (100%) and humerus (76.2%) fractures, respectively. It had low sensitivity in detection of in intra-articular extremity bone fractures. 
